# Selective serotonin reuptake inhibitors for functional independence and depression prevention in early stage of post-stroke: A meta-analysis

**DOI:** 10.1097/MD.0000000000019062

**Published:** 2020-02-07

**Authors:** Shaojiong Zhou, Shuo Liu, Xiaoqiang Liu, Weiduan Zhuang

**Affiliations:** a Shantou University Medical College; b Neurology Department, First Affiliated Hospital of Shantou University Medical College, Shantou, Guangdong, China.

**Keywords:** depression, functional independence, meta-analysis, selective serotonin reuptake inhibitors, stroke

## Abstract

**Background:**

The efficacy and safety of selective serotonin reuptake inhibitors (SSRIs) for functional independence and depression prevention in early stage of post-stroke (within 1 month after stroke onset) are still unclear.

**Methods:**

Relevant randomized controlled trials (RCTs) comparing early SSRIs therapy with placebo were sought from PubMed, Cochrane Library, Medline, and Embase. Primary outcomes were functional independence and depression occurrence. Secondary outcomes contained the improvement of Fugl-Meyer motor scale (FMMS) score and adverse events. We used fixed or random effects model to pooled effect estimates. And we chose risk ratio (RR) or mean differences (MDs) with the 95% confidence intervals (CIs) for data analysis.

**Results:**

We included 10 RCTs with total 5370 patients. The outcome of functional independence showed no significant difference between SSRIs and placebo group (RR, 1.28; 95% CI, 0.96–1.72; *P* = .10; *I*^2^ = 92%). However, depression occurrence differed significantly between these 2 groups, which favored SSRIs group (RR, 0.78; 95% CI, 0.67–0.90; *P* = .001; *I*^2^ = 23%). In addition, we observed that the side effects of SSRIs were seizure and nausea. Except psychiatric disorders/insanity rate was less in SSRIs group than placebo group (RR, 0.66; 95% CI, 0.48–0.90; *P* = .009) (*I*^2^ = 0%), other adverse events were revealed non-significant in our meta-analysis.

**Conclusions:**

Our meta-analysis revealed that early SSRIs therapy were effective to prevent post-stroke depression. However, SSRIs did not improve patient's post-stroke functional independence. In addition to increase the occurrence of seizure and nausea, SSRIs were relatively safe.

## Abstract 2

Supplemental Digital Content is available in the text

## 1 Introduction

With an increasing and aging population, the incidence of stroke grows rapidly. Stroke affects 43 million people of the world's population in 2015, and nearly 6.5 million of them have long-term disability.^[1]^ One-thirds of patients develop any kind of depression from 2 days to 7 years after stroke.^[2,3]^ When the benefits of intravenous thrombolysis and endovascular treatment for the prognosis of stroke are highly concerned and have been proved,^[4–6]^ a medical therapy for neurological functional recovery after stroke is also important and urgent to be resolved. Selective serotonin reuptake inhibitors (SSRIs) are a kind of drug for treating post-stroke depression,^[7]^ anger proneness and emotional incontinence.^[8,9]^ Their efficacy to alleviate post-stroke depression are well detected while the efficacy for post-stroke depression prevention are unclear.^[10]^ SSRIs are also observed to improve neurological functional recovery after stroke in animal model.^[11,12]^ Previous trials also showed that SSRIs can promote and redistribute the activation of motor cortex in post-stroke patients, which was associated with the improvement of motor performance.^[13,14]^ These findings enlightened a deep insight into the potential efficacy of SSRIs in the post-stroke population.

Previously, the efficacy and safety of SSRIs in post-stroke population had been studied in many relevant randomized controlled trials (RCTs). And a Cochrane review, written by Mead, concluded that SSRIs seemed likely to improve functional independence and prevent post-stroke depression.^[10]^ A trial examining the efficacy between early and late SSRIs therapy discovered that a better recovery of post-stroke impairment in the early SSRIs group. This trial indicated a better neurological functional recovery if SSRIs was administered early.^[15]^ In the past 2 decades, several RCTs, with small sample sizes, revealed various results on the efficacy of early SSRIs therapy.^[16–23]^ And a recent meta-analysis, written by Gu, affirmed that early SSRIs therapy was effective for ameliorating the modified Rankin Scale (mRS) score and the National Institutes of Health Stroke Scale (NIHSS) score after stroke, without efficacy for reducing the depression occurrence.^[24]^ However, opposite results were found in other new trials (FOCUS and TALOS) with larger sample sizes.^[25,26]^ Therefore, the potential efficacy of early SSRIs therapy for post-stroke functional independence and post-stroke depression prevention are still unclear. We aimed to perform a meta-analysis of the latest RCTs to assess the efficacy and safety of early SSRIs therapy for post-stroke functional independence and post-stroke depression prevention.

## 2 Materials and methods

We performed a meta-analysis according to the Cochrane Handbook for Systematic Reviews of Interventions.^[27]^ The detailed protocol of this meta-analysis was based on the Preferred Reporting Items for Systematic Reviews and Meta-Analyses (PRISMA) standards (dx.doi.org/10.17504/protocols.io.9qnh5ve [PROTOCOL DOI]).^[28]^ Supplemental table, demonstrates the PRISMA Checklist. We obtained ethical approval from the ethics committee of first affiliated hospital of Shantou University Medical College.

### 2.1 Literature search

Two authors systematically and independently searched literature published in English using 4 electronic databases (PubMed, Medline, Cochrane Library, and Embase) from inception to March 18, 2019. The keywords were as follows: ((((((stroke) OR cerebrovascular accident∗) OR cerebrovascular disorder∗) OR brain vascular accident∗) OR brain ischemi∗) OR brain hemorrhag∗) AND ((((((((((((((((serotonin uptake inhibitor) OR serotonin uptake inhibitor∗) OR citalopram) OR celexa) OR escitalopram) OR lexapro) OR fluoxetine) OR prozac) OR paroxetine) OR paxil) OR pexeva) OR sertraline) OR zoloft) OR vilazodone) OR viibryd) OR fluvoxamine). No other limitation of search was applied.

### 2.2 Study selection

The inclusion criteria were as following:

(1) recruitment of stroke patients (≥18 years old; onset <1 month), who were diagnosed ischemic stroke or intracerebral hemorrhage in accordance with brain imaging features;
(2) administration of SSRIs within 1 month after stroke onset;
(3) placebo treatment as comparison group;
(4) including one or more of the following outcomes: the mRS score for functional independence^[29]^; depression occurrence; the Fugl-Meyer motor scale (FMMS) score for motor recovery after stroke^[30]^; adverse events including insomnia, nausea, abdominal pain/stomachache, drowsiness/somnolence, sweating, dizziness, sexual dysfunction, psychiatric disorders/insanity, seizure, cardiovascular events, bleeding events and death;
(5) RCTs.

Two authors independently removed duplicated reports and manually identified irrelevant articles by titles and abstracts. Potentially relevant articles were evaluated by reading the full text. Any disagreement was resolved by consensus. Finally, the articles according with the inclusion criteria were remained for data extraction.

### 2.3 Data extraction

Two independent reviewers extracted data and entered them into standardized spreadsheets. The following study characteristics were collected from each included RCT: trial name, publication year, number of patients, mean age, gender, classification of stroke, the initiation time of intervention, detail of treatment, relevant outcomes/criteria, period of follow-up and results. Any data discrepancy was resolved by consensus.

### 2.4 Definition of outcomes

The primary outcomes were the rate of functional independence (mRS Score 0–2) and depression occurrence. Secondary outcomes included the improvement of FMMS score for motor functional recovery. And adverse events including insomnia, nausea, abdominal pain/stomachache, drowsiness/somnolence, sweating, dizziness, sexual dysfunction, psychiatric disorders/insanity, seizure, cardiovascular events, bleeding events and death.

### 2.5 Quantitative data synthesis and analysis

The process of data analysis was conducted by Review Manager 5.3.3 and Stata 12 software. The functional independence, depression occurrence, and adverse events were treated as dichotomous data. We calculated the risk ratio (RR) with the 95% confidence intervals (CIs) between SSRIs group and placebo group. The motor functional recovery was represented as continuous variables, and we calculated the mean differences (MDs) with 95% CIs. The heterogeneity between trials was analyzed using the *I*^2^ statistics and chi-squared (*Q*) according to Cochrane handbook. *I*^2^ < 40% indicates no heterogeneity, while I^2^ > 75% indicates considerable heterogeneity. 30%≤*I*^2^ ≤ 60% indicates moderate heterogeneity while 50%≤*I*^2^≤90% indicates substantial heterogeneity.^[27]^ The fixed-effects model was performed when *I*^2^ < 40%. When *I*^2^≥40%, sensitivity analyses were performed to reduce heterogeneity. If heterogeneity still existed, we chose the random effects model for data analysis. Subgroup analyses were performed based on the publication years (≥2018 vs <2018), sample sizes (≥200 vs <200), type of SSRIs (fluoxetine vs sertraline) and type of stroke (ischemic vs unspecified) to evaluate the various factors of the functional independence outcome. We also performed subgroup analyses by the outcome of depression occurrence based on the type of SSRIs (sertraline vs others), sample sizes (≥200 vs <200) and intervention course (>3 months vs ≤3 months). A *P* value <.05 was considered as significantly statistical difference. Publication bias assessment was completed by Begg test and Egger test.^[31,32]^

## 3 Result

### 3.1 Search result

**Figure 1:**
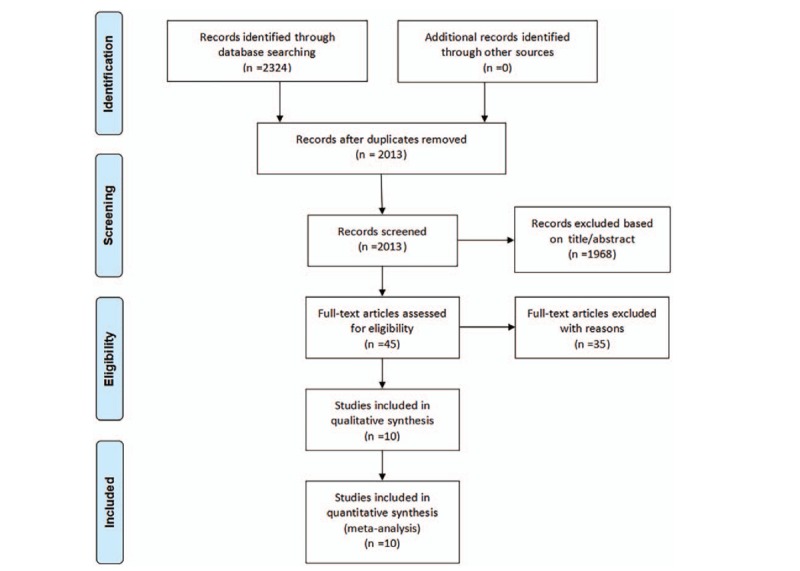
Flow diagram of literature search and study selection.

A total of 2324 articles were found after initial electronic search. After removed duplicates and irrelevant articles according to the titles and abstracts, 45 articles remained. After reviewing the full texts, ten RCTs met the inclusion criteria.^[17–23,25,26,33]^ The process of literature search and selection was summarized in a flow diagram (Fig. 1).

### 3.2 Characteristics of included studies

The characteristics of included studies were detailed in Table 1. The included studies contained a total of 5370 patients, among whom 2755 patients were randomized in the SSRIs group and 2615 patients in the placebo group. The trials were published from 2003 to 2018. The trials sample sizes ranged from 90 to 3127. The mean age and gender proportion were similar in each group. Among these trials, 3 trials included both ischemic stroke and hemorrhagic stroke patients, 1 trial did not specify the type of stroke, others only included ischemic stroke patients. In addition, 4 trials compared fluoxetine with placebo, 2 trials compared citalopram with placebo, 2 trials compared sertraline with placebo, 1 trial compared escitalopram with placebo, and 1 trial compared fluoxetine and citalopram with placebo. The end points of functional independence and depression occurrence were analyzed in 4 trials respectively.

**Table 1:**
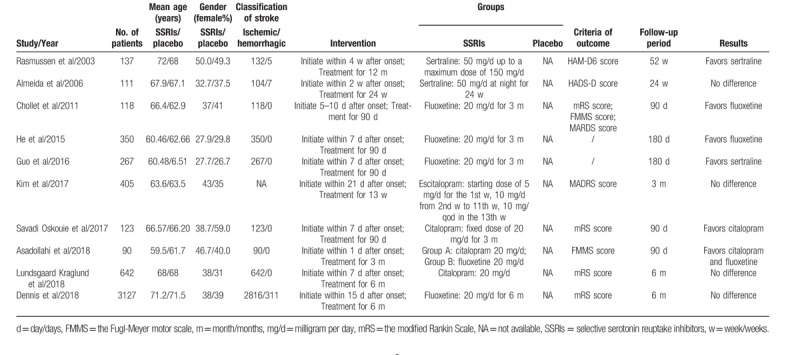
Characteristics of included studies.

### 3.3 Risk of bias

**Figure 2:**
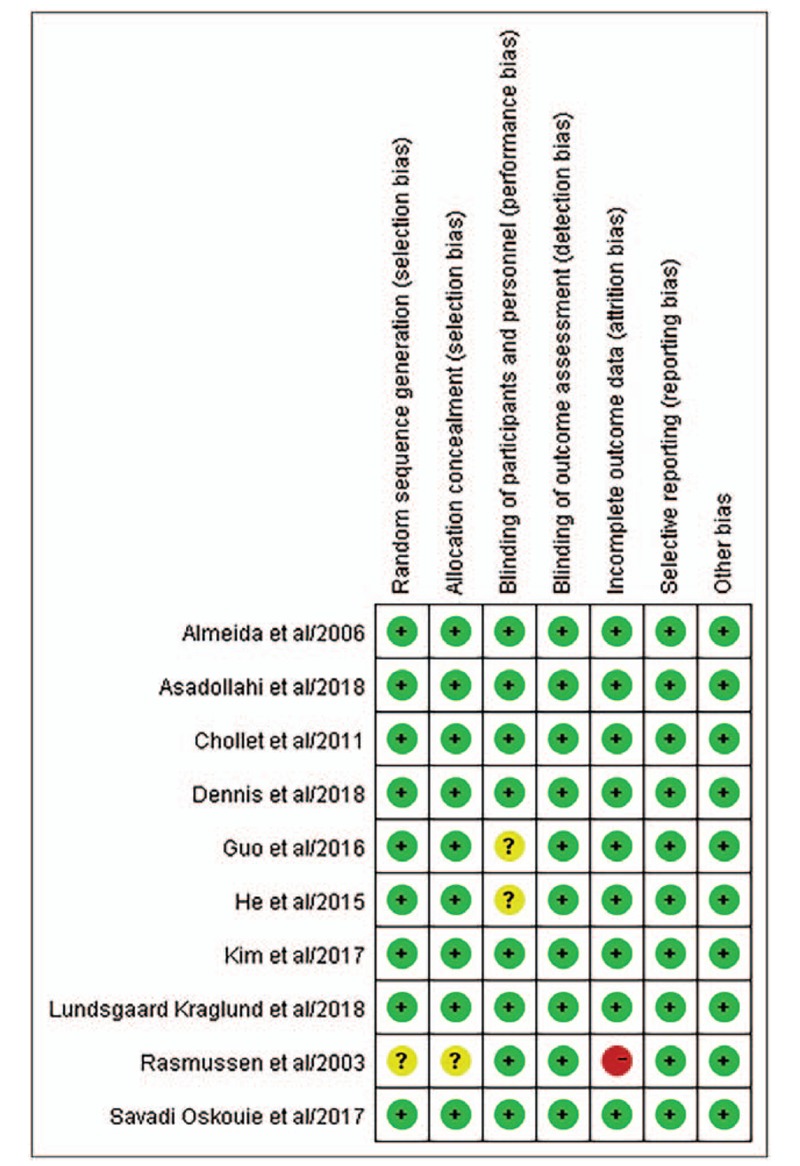
Risk of bias summary. Green indicates low risk; yellow indicates unclear risk; red indicates high risk.

According to Cochrane Handbook for Systematic Reviews of Interventions, 2 reviewers independently assessed the risk of bias: random sequence generation, allocation concealment, blinding of participants and personnel, blinding of outcome assessment, incomplete outcome data, selective reporting. Any disagreement was resolved by consensus. Overall, 2 of included trials did not demonstrate the blinding of participants and personnel clearly,^[20,21]^ which indicated unclear risk. Another trial, which indicated high risk of bias, did not detailed the random sequence generation, the allocation concealment and the incomplete outcome data. Details are shown in Figure 2.

### 3.4 Primary outcomes

**Figure 3:**
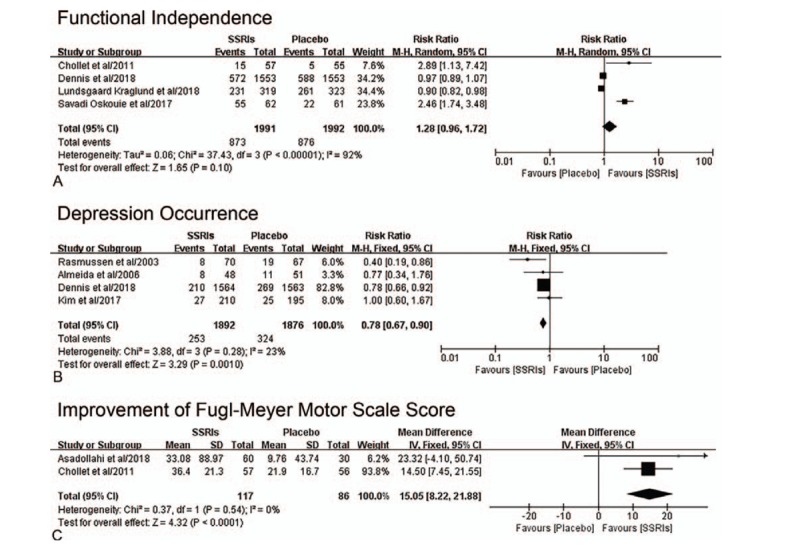
Forest plot of outcomes of early SSRIs therapy compared with placebo therapy. [A] Functional independence (the modified Rankin scale Score 0-2) from the baseline to the end of follow-up (90 days,^[18,19]^ 6 months^[25,26]^). The modified Rankin scale measures functional independence outcome on a 7-point ordinal scale: 0, no symptoms at all; 1, no significant disability despite symptoms; 2, slight disability; 3, moderate disability; 4, moderately severe disability; 5, severe disability; 6, death. [B] Depression occurrence from the baseline to the end of follow-up (3 months,^[17]^ 24 weeks,^[22]^ 6 months,^[26]^ 52 weeks^[23]^). [C] The improvement of Fugl-Meyer motor scale score from the baseline to the end of follow-up (90 days^[18,33]^). SD = standard deviation, CI = confidence interval, SSRIs = selective serotonin reuptake inhibitors.

The data of functional independence (mRS score 0–2), which indicated good functional outcome,^[3,29]^ was offered in 4 trials, including 3983 patients. No significant difference was observed between SSRIs group and placebo group (RR, 1.28; 95% CI, 0.96–1.72; *P* = .10) (Fig. 3A). Due to the existing high heterogeneity (*I*^2^ = 92%), we conducted sensitivity and subgroup analyses. Sensitivity analyses showed the consistent outcome. As for subgroup analyses, significant difference was detected in the trials having smaller sample (n < 200) and publishing before 2018 (RR, 2.51; 95% CI, 1.81–3.47; *P* < .001; *I*^2^ = 0%). And the considerable subgroup differences (*I*^2^ = 96.9%) was observed. Other subgroup analyses (fluoxetine vs citalopram; ischemic stroke versus unspecified) revealed the similar statistic outcomes. Table 2 showed the details.

**Table 2:**
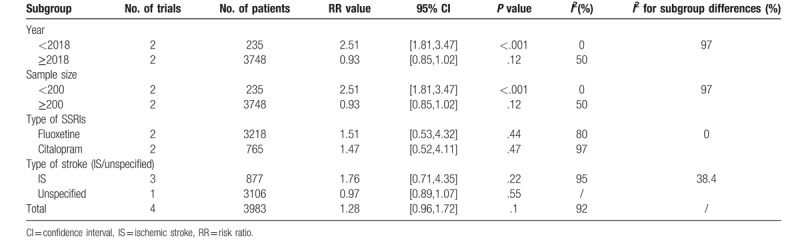
Subgroup analysis for functional independence.

Four trials, including 3768 patients, provided data of depression occurrence. The occurrence of depression differed significantly between SSRIs group and placebo group, which favored SSRIs group (RR, 0.78; 95% CI, 0.67–0.90; *P* = .001) with low heterogeneity (*I*^2^ = 23%) (Fig. 3B). Inconsistent result was found in subgroup with a shorter course of treatment (≤3 months) (RR, 1.00; 95% CI, 0.60–1.67; *P* < .99; Subgroup differences: *I*^2^ = 8.2%). Table 3 showed the details.

**Table 3:**
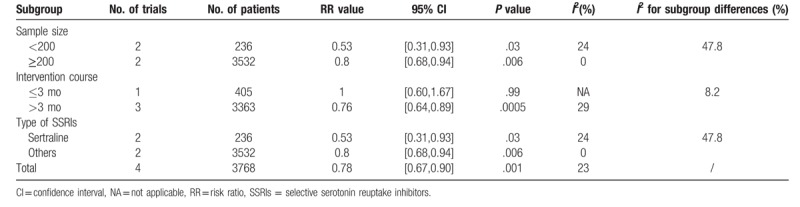
Subgroup analysis for depression occurrence.

### 3.5 Secondary outcomes

**Figure 4:**
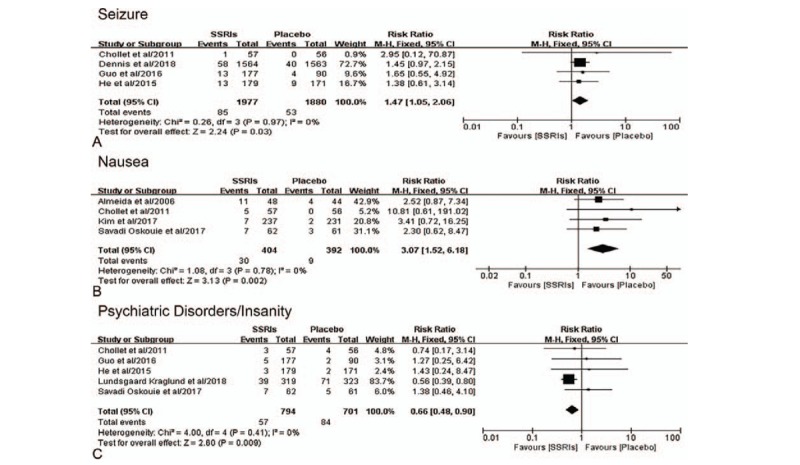
Forest plot of adverse events of SSRIs therapy compared with placebo therapy. [A] Seizure. [B] Nausea. [C] Psychiatric disorders/insanity. CI = confidence interval, SSRIs = selective serotonin reuptake inhibitors.

The patients allocated SSRIs had a greater improvement of FMMS score (MD, 15.05; 95% CI, 8.22–21.88; *P* < .001) (*I*^2^ = 0%) than those allocated placebo (Fig. 3C). In addition, adverse events about seizure (RR, 1.47; 95% CI, 1.05–2.06; *P* = .03) (*I*^2^ = 0%) and nausea (RR, 3.07; 95% CI, 1.52–6.18; *P* = .002) (*I*^2^ = 0%) had significant difference between these 2 groups (Fig. 4A, B). The patients allocated SSRIs were less likely to have psychiatric disorders/insanity (RR, 0.66; 95% CI, 0.48–0.90; *P* = .009) (I^2^ = 0%) than those allocated placebo (Fig. 4C). Other adverse events, including insomnia, abdominal pain/stomachache, drowsiness/somnolence, sweating, dizziness, sexual dysfunction, cardiovascular events, bleeding events, and death, showed no significant difference between SSRIs group and placebo group (see Supplemental figure. Figure shows other adverse events of SSRIs therapy compared with placebo therapy).

### 3.6 Publication bias

**Figure 5:**
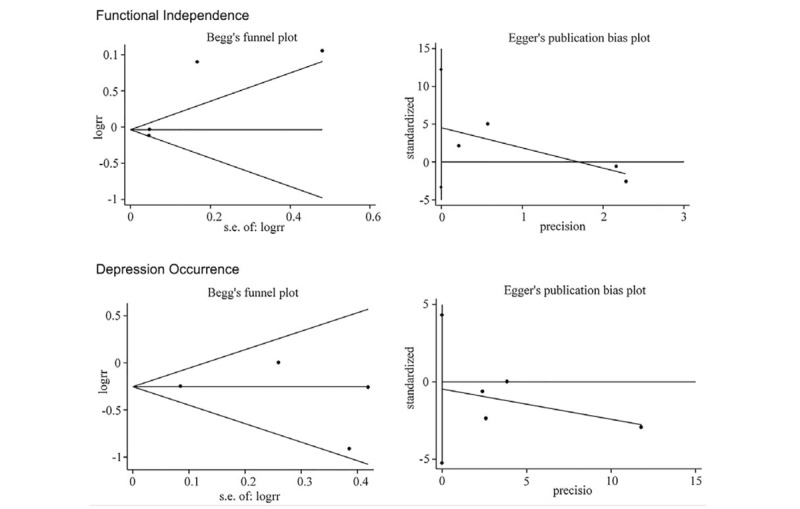
Publication bias plot of primary outcomes. [A] Functional independence. [B] Depression occurrence.

For functional independence, no evidence of publication bias was revealed (Egger test, 0.129; Begg test, 0.308). And the same result was observed in the outcome of depression occurrence (Egger test, 0.715; Begg test, 0.734) (Fig. 5).

## 4 Discussion

We performed a meta-analysis of ten trials to evaluate the efficacy and safety of early SSRIs therapy for post-stroke functional independence and post-stroke depression prevention. Firstly, the outcome of functional independence did not differ significantly between SSRIs group and placebo group. And a part of subgroup analyses (fluoxetine vs citalopram; ischemic stroke vs unspecified) revealed the similar statistic outcomes. On the contrary, other subgroup analyses (publication years ≥2018 vs <2018; sample sizes ≥200 vs <200) showed significant statistic difference. And a considerable subgroup differences was also detected. Secondly, early SSRIs therapy was associated with the lower occurrence of post-stroke depression and the improvement of FMMS score. In addition, the assessment of adverse events showed a significantly higher rate of seizure and nausea in patients treated with SSRIs than those treated with placebo. And we found that SSRIs was also related to a lower occurrence of psychiatric disorders/insanity after stroke.

The mRS score, as a valid and practical criteria of post-stoke functional outcome, provides a reliable method to assess the functional independence after stroke.^[34,35]^ The primary outcome of our meta-analysis showed that early SSRIs therapy did not significantly improve patient's functional independence, which was opposite to the result of a meta-analysis written by Gu.^[24]^ Our meta-analysis was the first 1 primarily to analyze the efficacy of early SSRIs therapy compared with placebo for functional independence and obtained a negative result. We included two latest RCTs (FOCUS and TALOS),^[25,26]^ which had much larger sample sizes and longer period of follow-up. So we believe that our result is more reliable than the previous meta-analysis.

According to the result of depression occurrence and adverse events, we determined that early SSRIs therapy can effectively reduce the occurrence of post-stroke depression and increase seizure and nausea events, which indicated SSRIs was an effective prophylactic treatment for post-stroke depression but had adverse effects of seizure and nausea. These results were also opposite to the results of the meta-analysis written by Gu and consistent with another previous meta-analysis written by Yi.^[24,36]^ More trials with a much larger number of patients were included in our meta-analysis and a great homogeneity (depression occurrence: *I*^2^ = 23%; seizure event: *I*^2^ = 0%; nausea event: *I*^2^ = 0%) was observed. Therefore, we believe that our findings are much more reliable. In addition, our meta-analysis was the first 1 to find that early SSRIs therapy was related to a lower occurrence of psychiatric disorders/insanity after stroke. It might indicate that SSRIs is an effective medicine for post-stroke psychiatric disorders/insanity. We think SSRIs is relatively safe in post-stroke population except the side effect like seizure and nausea. A comprehensive assessment needs to be considered by physician in clinical practice.

Early SSRIs therapy also improved FMMS score, which indicated the recovery of motor performance. Similarly, the part of motor function in NIHSS was also statistically significant between SSRIs and placebo group in the FLAME trial,^[18]^ while no difference was observed in other parts of NIHSS. Besides, as we mentioned above, previous trials also showed that SSRIs improve motor performance in post-stroke patients based on the mechanisms of promoting and redistributing the activation of motor cortex.^[13,14]^ Therefore, Early SSRIs therapy do not improve post-stroke functional independence, but it seems that Early SSRIs therapy can specifically improve post-stroke motor performance. More reliable studies were required to testify.

Some limitations, including the limited participants number (<200 in each trial), short follow-up (<90 days mostly) was addressed and reported in the meta-analysis written by Gu.^[24]^ Two latest trials (FOCUS and TALOS) included in our meta-analysis contained much larger number of participants and longer follow-up time, which enhanced the statistical power in our meta-analysis. And the longer follow-up time (6 months) was more reliable for the long-term assessment of stroke recovery and post-stroke functional independence. Differences between previous meta-analyses and ours are showed in Table 4.

**Table 4:**
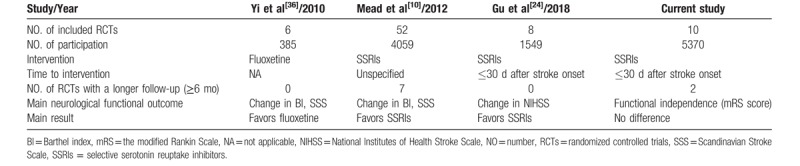
Comparison with previous meta-analyses.

Our meta-analysis had several limitations. On the one hand, it was unable to accurately detect the safety of SSRIs due to the limited data of adverse events, especially cardiovascular events, bleeding events and death. On the other hand, the trial FOCUS included a few proportions of depression patients. It was not very reasonable to analyze the outcome of post-stroke depression occurrence, although it had an undifferentiated baseline and we only extracted the data of new depression patients after intervention.

In conclusion, early SSRIs therapy could not improve patient's post-stroke functional independence. But it can effectively reduce post-stroke depression occurrence, indicating its efficacy of preventing post-stroke depression. In addition to increase the occurrence of seizure and nausea, SSRIs are relatively safe and effective to reduce post-stroke depression and psychiatric disorders/insanity. Most of previous studies made a conclusion that SSRIs was effective for stroke recovery.^[10,18,19,24]^ However, the negative result for post-stroke functional independence in our meta-analysis enlightens our suspect of the efficacy of early SSRIs therapy for neurological functional recovery. With the improving recognition of the negative impact of post-stroke depression, our findings can provide physician a valuable proof of post-stroke depression prophylactic treatment and even might update present clinical guideline, although the opinion of routinely initiate SSRIs therapy as early as we can to prevent post-stroke depression is not well accepted now. The results of ongoing trials are greatly concerned to provide more reliable evidence for the efficacy and safety of early SSRIs therapy.^[37]^

## Author contributions

**Conceptualization:** Weiduan Zhuang.

**Data curation:** Shaojiong Zhou, Shuo Liu.

**Formal analysis:** Shaojiong Zhou.

**Methodology:** Shaojiong Zhou, Shuo Liu, Xiaoqiang Liu.

**Project administration:** Xiaoqiang Liu, Weiduan Zhuang.

**Software:** Shaojiong Zhou, Shuo Liu.

**Supervision:** Xiaoqiang Liu, Weiduan Zhuang.

**Writing – original draft:** Shaojiong Zhou, Shuo Liu.

**Writing – review & editing:** Shaojiong Zhou, Shuo Liu, Weiduan Zhuang.

## Supplementary Material

Supplemental Digital Content

## Supplementary Material

Supplemental Digital Content
