# Supplementary material for: Selective serotonin reuptake inhibitors for functional independence and depression prevention in early stage of post-stroke: A meta-analysis
Source: Medicine (Baltimore). 2020 Feb 7;99(6):e19062. doi: 10.1097/MD.0000000000019062 (PMC7015581; doi:10.1097/MD.0000000000019062)

**Supplemental figure:** Forest plot of other adverse events of SSRIs therapy compared with placebo therapy, including insomnia, abdominal pain/stomachache, drowsiness/somnolence, sweating, dizziness, sexual dysfunction, cardiovascular events, bleeding events, and death. Abbreviations: CI, confidence interval; SSRIs, selective serotonin reuptake inhibitors.


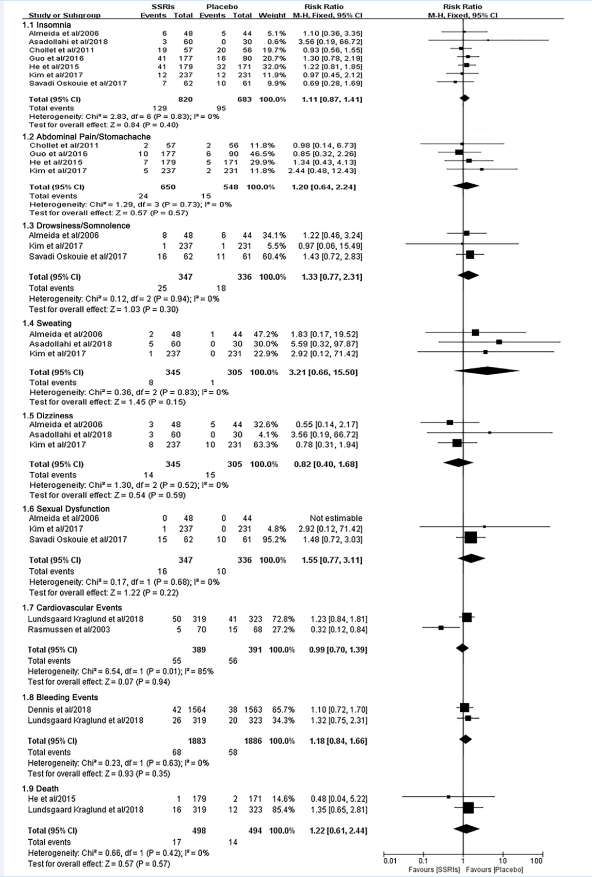

Supplement: Supplemental Digital Content [file medi-99-e19062-s001.doc]
